# The Sleep of Shelter Dogs Was Not Disrupted by Overnight Light Rather than Darkness in a Crossover Trial

**DOI:** 10.3390/ani9100794

**Published:** 2019-10-14

**Authors:** Katherine A. Houpt, Hollis N. Erb, Genaro A. Coria-Avila

**Affiliations:** 1 Department of Clinical Sciences, Cornell University, Ithaca, NY 14853, USA; 2 Population Medicine and Diagnostic Sciences, Cornell University, Ithaca, NY 14850, USA; hne1@cornell.edu; 3 Centro de Investigaciones Cerebrales, Universidad Veracruzana, Xalapa-Enríquez 91090, Mexico; gcoria@uv.mx

**Keywords:** sleep, dog, shelter, influence of light, night-time behavior

## Abstract

**Simple Summary:**

Dogs are often left unattended overnight in shelters. This has led shelter mangers to worry that the dogs were suffering from separation anxiety and, therefore, exhibiting stereotypic or repetitive behaviors such as pacing, barking or digging at exits. Mangers also worried that dogs exposed to light at night were not able to sleep or slept less because some areas of the shelter were lit for security reasons. The ten dogs in this study were walked twice daily and provided with many enrichment devices and music during the day. They were housed in individual rooms each with a window allowing visualization of the dog by the public and vice versa. The dogs slept 10.8 h/night when in darkness and 10.5 h/night when their pens were lightened; there was no significant difference. They exhibited no stereotypic behaviors. They slept in bouts of slightly less than an hour, arising after each bout to stand up and then lie down again. Apparently, these well-stimulated shelter dogs slept soundly in the absence of people.

**Abstract:**

Dogs in shelters may be unattended at night. The purpose of this study is to describe the night-time behavior of dogs in a shelter and to determine if artificial light affected their sleeping patterns. Ten dogs were video-recorded under both light and dark conditions and their behavior recorded using focal animal sampling. The dogs were lying down 649 ± 40 min (mean ± SD) in the light condition and 629 ± 58 min in the dark condition each night. They awoke, stood up, turned around and then lay down again every 48 to 50 min. There was no significant difference in time spent lying between the two conditions (*p* > 0.05). Light did not seem to affect their behavior. The conclusion is that dogs in shelters may sleep in the absence of people and that light does not disrupt their sleep patterns.

## 1. Introduction

Some of the behaviors of dogs living in kennels in shelters could be considered as abnormal. Those are, for example: excessive barking, excessive pacing, and self-mutilation [1]. Although three million dogs are relinquished to shelters each year [2] there have been few studies of the dogs’ behavior when the shelters are closed (that is, at night). Because some dogs will be pacing, jumping at the door, and vocalizing when the staff arrives in the morning, the fear has been that the dogs may be spending the night in repetitive behaviors. In addition, some of the shelter’s kennels were illuminated at night from lit hallways. This study was undertaken to determine how the animals spent their time at night and whether they were disturbed if their kennel was lit at night.

To analyze dogs’ behavior and welfare it is important to know their sleep duration and patterns as well as their behavior while awake. Pet dogs living in a home show diminished activity between 11:00 p.m. and 6:00 a.m. [3]. Owned dogs sleep for 8 h in 23 sleep-wake cycles of 16 min asleep and 5 min awake [4]. Jones et al. [5] used accelerometers to measure activity of shelter dogs and found that stress as measured by cortisol levels and behavior signs based on the dog’s posture, tail and ear position and vocalization etc. varied directly with activity; the more active dogs were more stressed. There is only one study reporting on the sleep of dogs living in an animal shelter [6], but the lighting was not described.

Humans exposed to light during the night can experience a variety of health problems including an increased risk of cancer, metabolic syndromes, and more rapid aging [7,8]; but these might be impacted by individual preferences. Domestic dogs might suffer from these disorders if exposed to artificial light. Shelters may have lights that cannot be extinguished for security or other reasons, which might provoke disruptions in sleep or excessive barking, pacing around the kennel, jumping, etc.

The objective of this experiment was to describe the night-time behavior of dogs in shelters and the influence of artificial light. Our hypothesis was that the lack of people or the presence of light affects sleep patterns in dogs. Our prediction was that the dogs would exhibit night-time behavior similar to that of pet dogs.

## 2. Methods

This study was approved by the Institutional Animal Care and Use Committee of Cornell University (2008-0094).

### 2.1. Subjects

The population studied consisted of 10 dogs (four males and six females). Two of the females were spayed 4 d before observations began and one male was castrated 13 d before observations began. All the dogs were eventually adopted within a month of the observations, except for one dog that was adopted 5 months later. The age range was from 6 months to 7 years (mean 2 years). Seven dogs were Pit Bulls, two Shepherds and one a Labrador mix. They were kept at the Society for the Prevention of Cruelty to Animals of Tompkins County in the adoption center. Each dog was kept individually in a room 4.32 m by 6.96 m. The walls and floor were cement. Three walls were solid and the front wall had a window and a door. The dogs could not see one another from their rooms. Each room contained a raised bed consisting of canvas supported by a PVC frame (Kuranda^®^, Glen Burnie, Md, USA) and covered with a quilt or blanket. Food and water bowls were stainless steel.

### 2.2. Management

#### 2.2.1. Daily Schedule

From 08:00 h to 08:30 h the dogs were taken for a walk outside and then fed. Once they had finished they were taken to a different room while their assigned one was cleaned. At noon they received a second meal. In addition to the early morning walk volunteers walked the dogs outside twice a day for 30 min.

#### 2.2.2. Environmental Enrichment

Classical music, which was found to reduce signs of stress in shelter dogs [9], was played in each room from noon to closing time at 17:00. Animals were provided with food-toys twice a week; among these were cardboard egg cartons filled with treats, soft boxes filled with treats inside other boxes (forcing them to dig to find the reward), and a metal soup can filled with dog food and treats. Volunteers gave the dogs Kongs^®^ (Cuyahoga Falls, OH, USA) filled with peanut butter and treats each day before closing.

### 2.3. Experimental Design and Statistical Analysis

#### 2.3.1. Light vs. Dark

The timing of treatments was balanced so that half the dogs were filmed first in the light provided by a standing lamp with a 200-watt bulb (3000 lumen), or by florescent light (2000 lumens) which could not be extinguished in an adjacent hallway and the other half first in the dark (no light source in a windowless building). Each trial lasted 12 h (19:00 to 07:00) while humans were not in the environment. To record behavior a SPECO technologies infrared camera model cvc325wp5 was used, as well as a Panasonic VCR model AG 6124. The recordings were made at 1/6 normal speed so that 12 h could be recorded on a 2-h cassette. The study was carried out during February and March, 2009.

#### 2.3.2. Sampling Method

The behaviors were recorded using an instantaneous scan sample at 1 min intervals for a 12 h period [10]. Time spent in each behavior in the light and dark conditions was measured by a single observer: a graduate student in behavior who had performed observational studies on dogs previously.

The behaviors observed during the experiment were: sleep (head down in lateral or sternal recumbency), bout length (time spent sleeping), bout number (the number sleeping bouts per night), the number of turns and walking (see Table 1 for definitions). Stereotypic behaviors such as pacing, spinning, jumping on walls, door or window, barking for more than 5 min at a time, digging at the floor or bed and self mutilation were to be recorded.

#### 2.3.3. Statistical Analyses

A Shapiro-Wilk test was used to test for normality of the paired, within-dog differences. Because the data were Gaussian a paired t-test was used to compare frequency differences. Statistix^®^ (Tallahassee, FL, USA) was the program used. Alpha level was set at *p* < 0.05. The results are presented as mean and Standard Deviation (SD).

## 3. Results

The most common behavior was recumbency which occupied the majority of the 720 min of observations/night. The dogs were lying down 649 ± 40 min (mean ± SEM) in the light condition and 629 ± 58 min in the dark condition each night. They awoke, stood up, turned around and then lay down again every 48 to 50 min. There was no significant difference in time spent lying between the two conditions (*p* > 0.05). The dogs had many sleeping bouts/night under artificial light conditions and under darkness. The length of these bouts was less than an hour under both artificial light conditions and under darkness. No significant differences were found in sleep length or bout number or length between the dark and the light (*p* > 0.05 paired t-test) (see Table 2). The dogs never lay down off their beds. No stereotypic behaviors were observed under either condition.

## 4. Discussion

These shelter dogs slept from the time humans left the shelter until shortly before they returned in the morning. The dogs did not show stereotypic behavior, but became active just before the personnel returned in the morning. During the experiment we expected to find that artificial light would disturb the dogs’ sleep but found instead that there was neither a difference in total sleep time nor in the number and length of each bout of sleeping. Dogs were asleep 649 min of a 12 h period at night under artificial light and 629 min in darkness—a 20 min, non-significant difference that we suspect is also of no practical importance to the dogs’ well-being across a span of 720 min. Therefore, in this kennel artificial light did not influence sleep.

One of the most interesting findings was that the dogs slept in bouts of almost an hour and turned around before lying down again. We have previously reported on another group of dogs in the same environment to determine the direction in which the dogs curled and the side on which they lay during each bout [11]. The dogs did not exhibit laterality and in most cases changed sides after arising.

The limitations of this study are the small number of dogs (*n* = 10) in only one shelter and the lack of intra-observer accuracy test. All the dogs were medium to large dogs; smaller dogs may exhibit different sleeping patterns. It is difficult to distinguish whether a recumbent dog is asleep or awake, although the tapetal reflection is obvious when the dogs were video-recorded in the dark. A few dogs were observed soon after neutering which may have affected their sleep time, but their behavior seemed very similar to the other dogs. For example, one recently spayed bitch, Latte, slept 673 min in the light while the range was 576–697 min. She slept 671 min in the dark while the range was 499−684 min. Her sleep time was within the range of all the dogs under both illumination conditions.

The dogs slept in 13 to 14 bouts per night of 48 to 50 min in length under each of the two conditions during the 12 h period of observation from 19:00 h to 07:00 h. The average sleeping bout length was 48 min under artificial light and 50 min in darkness. Adam and Johnson [4] video-recorded owned dogs in their homes for 8 h and found that they slept in 23 bouts of 16 min separated by 5 min of wakefulness. As compared to the results of this study it indicates that dogs sleeping with humans have more bouts, and less total sleep time at night. Thus, they do not sleep as soundly when sharing their environment with humans. However, another possible explanation is that the household pets studied by Adams and Johnson were not exercised or stimulated as much as the shelter dogs. The level of physical activity may affect sleep length. Adams and Johnson also found similar patterns of 3 sleep-wake sessions/h in detector dogs and in guard dogs [12]. One study showed that laboratory beagles slept only 1 to 5 min/25 min of daytime observation, most in a cage and least in an indoor run [13]. Furthermore, when recording for 8 h during the day, Hite et al. [14] found that dogs in 30-in × 30-in cages lay down significantly less than dogs in 30-in × 90-in cages during the day. No recordings were made during the day in the present study. The large size of the enclosures in the present study may have contributed to the longer sleep bouts and total sleep time because the dogs were less disturbed.

In this study the dogs slept 11 of the 12 h that they were alone but the dogs did not sleep continuously. They displayed a pattern of awakening every hour, arising and turning around, usually changing the direction in which they curled. Shelter dogs were observed in the United Kingdom (UK) and a similar pattern was found, but the dogs had many more sleep bouts [6] The dogs had 33 sleep bouts at night each occupying 25 min whereas the dogs in the present study slept in 13 or 14 bouts of 48 to 50 min in length. Sleep occupied 71.5% of the night of the UK dogs and 87% to 90% of the night of the US dogs. The reason for the more fragmented sleep in the UK dogs is not clear. The UK dogs were housed in a much smaller kennel, 3.8 m × 2.2 m in comparison to the 4.32 m × 6.96 m room in which the US dogs were housed. The UK dogs had periodic access to an indoor run (2 m × 4 m), but probably not at night. Dogs living with their owners had 23 sleep bouts of 16 min (values midway between the two shelter studies) but they slept for only 8 h in contrast to the approximately 11 h nights for these 10 shelter dogs. Whether or not dogs sleep more in shelters than in homes, their corticosteroid levels fall when they are taken from a shelter to a home environment for one night [15].

Further studies of sleep patterns in dogs in shelters and other environments should be undertaken. Temperature and humidity might alter sleep. Sleep patterns may vary with types of lighting, opening hours and presence of staff after hours and other species housed in the shelter. These studies should include measurements of stress, such as corticosteroid levels, to determine if shorter sleep bouts or shorter total sleep time is associated with physiological indicators of stress.

## 5. Conclusions

Dogs in this shelter slept most of night, but awoke and turned around periodically. Their sleep was not interrupted by artificial light during the night.

## Figures and Tables

**Table 1:** Definition of behaviors.

Behavior (Units)	Definition
Sleep (minutes)	Dog lies in sternal or lateral recumbency
Turn (number)	Dog arises and turns clockwise or counterclockwise before lying down again
Walk (minutes)	Dog moves two or more steps off the bed
Sleep bout (minutes)	Time between lying and arising

**Table 2:** Sleep times of shelter dogs in light and darkness.

Behavior	Light	Dark	*t*	*p* Value (1-Sided)
Total minutes of sleep	648.7 ± 40	629.3 ± 57.9	0.92	0.81
Range	576–697	499–684		
Length of sleep bout (min)	47.9 ± 9	50 ± 35.6	0.58	0.71
Range	36–61	36–76		
Number of sleep bouts	14 ± 2.2	13.4 ± 35	0.58	0.29
Range	11–17	9–17		
Minutes turning around	17.7 ± 10	14.2 ± 5	1.13	0.14
Range	9–40	7–23		
Minutes walking	14.4 ± 23	14.9 ± 13.2	0.11	0.54
Range	1–80	1–42		

Values are means ± SD. all df = 9.
